# Metabolomic Profiles of Multidrug-Resistant *Salmonella* Typhimurium from Humans, Bovine, and Porcine Hosts

**DOI:** 10.3390/ani12121518

**Published:** 2022-06-10

**Authors:** Jessie M. Overton, Lyndsey Linke, Roberta Magnuson, Corey D. Broeckling, Sangeeta Rao

**Affiliations:** 1 Department of Clinical Sciences, College of Veterinary Medicine and Biomedical Sciences, Colorado State University, Fort Collins, CO 80523, USA; jessie.earhart@colostate.edu (J.M.O.); lyndsey.linke@colostate.edu (L.L.); magnusonroberta@gmail.com (R.M.); 2 Analytical Resources Core: Bioanalysis and Omics, Colorado State University, Fort Collins, CO 80523, USA; corey.broeckling@colostate.edu

**Keywords:** antimicrobial resistance, metabolomics, metabolites, *Salmonella* Typhimurium, resistance markers

## Abstract

**Simple Summary:**

The global threat that is imposed by the resistance the pathogens develop to antimicrobial drugs is escalating. Tools to detect the resistance (with evidence on molecular and cellular outcomes) would reveal intricate mechanisms through which novel drugs could be developed. Approaches such as metabolomics, which involve metabolite detection, provide scientific evidence of metabolite expression of antimicrobial-resistant pathogens. The current study involved metabolomics of antimicrobial-resistant *Salmonella* Typhimurium collected from various hosts (human, porcine, bovine) and were exposed to antimicrobial drugs—ampicillin, chloramphenicol, streptomycin, sulfisoxazole, and tetracycline—as one set of the experiment. The same isolates were also cultured with no drug exposure as a comparison. There are certain pathways of metabolite expression that are impacted by drug exposure when compared to no drug exposure, meaning that the expressed metabolites could be potential targets for drug companies for the treatment of antimicrobial-resistant pathogens.

**Abstract:**

Antimicrobial resistance (AMR) is a global public health threat, yet tools for detecting resistance patterns are limited and require advanced molecular methods. Metabolomic approaches produce metabolite profiles and help provide scientific evidence of differences in metabolite expressions between *Salmonella* Typhimurium from various hosts. This research aimed to evaluate the metabolomic profiles of *S.* Typhimurium associated with AMR and it compares profiles across various hosts. Three samples, each from bovine, porcine, and humans (total *n* = 9), were selectively chosen from an existing library to compare these nine isolates cultured under no drug exposure to the same isolates cultured in the presence of the antimicrobial drug panel ACSSuT (ampicillin, chloramphenicol, streptomycin, sulfisoxazole, tetracycline). This was followed by metabolomic profiling using UPLC and GC–mass spectrometry. The results indicated that the metabolite regulation was affected by antibiotic exposure, irrespective of the host species. When exposed to antibiotics, 59.69% and 40.31% of metabolites had increased and decreased expressions, respectively. The most significantly regulated metabolic pathway was aminoacyl-tRNA biosynthesis, which demonstrated increased expressions of serine, aspartate, alanine, and citric acid. Metabolites that showed decreased expressions included glutamate and pyruvate. This pathway and associated metabolites have known AMR associations and could be targeted for new drug discoveries and diagnostic methods.

## 1. Introduction

Antimicrobial resistance (AMR) in bacteria isolated from animal hosts is a major global public health threat. The Centers for Disease Control and Prevention have determined that AMR is “one of the greatest public health challenges of our time” [1]. One of the key goals for slowing or decreasing AMR identified by the White House in the National Strategy for Combatting Antibiotic-Resistant Bacteria is to “accelerate basic and applied research and development for new antibiotics…” [2]. However, current tools for detecting phenotypic resistance patterns are limited and require advanced molecular methods to reveal associations with AMR patterns [3,4].

Metabolomics is a relatively new tool that can be used to construct metabolite profiles and these metabolite patterns provide evidence of metabolite regulation at the cellular level. When bacteria are exposed to antibiotics, this exposure can trigger cellular changes within the bacterial cell that results in specific metabolic patterns that can help predict antimicrobial drug resistance profiles. Such predictability can have an immediate impact on human and animal health by leading to advancements in drug discoveries by targeting the expression of certain metabolites, as well as diagnostic tools to screen large numbers of samples for AMR. Identifying possible new drug targets would help pharmaceutical companies develop more specific and effective antibiotics to combat AMR bacterial infections [4].

Recent studies have shown that bacteria produce specific metabolic fingerprints when exposed to different classes of antibiotics. These fingerprints can help predict the mode of action used by antibiotics [4] to help develop any novel therapies. For many years, the development of antibiotic resistance could be partly explained by the synthesis of novel analogues of existing compounds [4]. However, such chemical modifications are finite, to keep pace with the remarkable adaptability of the bacteria when exposed to these selective drug pressures in the environment. To combat the prevalence of multidrug-resistant (MDR) pathogens, novel antibiotics that target distinct cellular functions are needed [5]. Better understanding the metabolic patterns in AMR bacteria to expose new cellular functions associated with drug resistance and susceptibility is one way to identify new drug targets. One of these studies also suggested that a core metabolic profile for each bacterium is identifiable regardless of the environmental condition, suggesting bacteria could be identified using in vitro metabolic profiles whether in a wound, on surgical equipment, or in the environment [6].

*Salmonella* is a rod-shaped, Gram-negative bacillus that belongs to the Enterobacteriaceae family. This organism is of high public health importance due to its ability to cause several syndromes in both animals and humans. Enteritis, septicemia, abortion, and asymptomatic miscarriages are the major syndromes that present in animals. Enteric fever, gastroenteritis, septicemia, and focal infections are the major syndromes that present in humans infected with *Salmonella* bacteria [7]. *Salmonella enterica* serovar Typhimurium is of utmost importance to public health due to its ability to infect human hosts via contaminated foods. It is one of the most identified serovars in cattle, humans, and pigs and has displayed resistance to ampicillin, chloramphenicol, sulfamethoxazole, and tetracycline [8].

The aim of this project was to identify cellular biomarkers (metabolites) associated with mechanisms of AMR in *Salmonella* Typhimurium using metabolomics and investigate the diversity of those markers among established genetic patterns of resistance in *S*. Typhimurium isolated from humans, bovine, and porcine samples. Metabolomics can be used as a tool to identify the cellular effects of AMR in this pathogen of public health importance. This research has two specific aims (1) to establish metabolomic profiles of *Salmonella* Typhimurium isolated from humans, porcine, and bovine and cultured in the presence and absence of an ACSSuT panel of drugs, and (2) to evaluate similarities and differences in these metabolomic profiles in *Salmonella* Typhimurium across isolates originating from humans, porcine, and bovine hosts. The hypothesis of the study is that non-targeted metabolite profiling will identify biomarker profiles distinctive of AMR in *S*. Typhimurium and, more specifically, the metabolite patterns will differ across various host species.

## 2. Materials and Methods

### 2.1. Isolate Collection, Screening, Identification, and Growth

*Salmonella* isolates from various institutes (*Salmonella* Typhimurium isolates were contributed by the Colorado Department of Public Health and Environment, CSU-Veterinary Diagnostic Laboratory, Ohio State University, University of Illinois, University of Pennsylvania, and Washington State University) in the US were shipped directly to the Animal Population Health Institute laboratory. A total of 88 human, 33 bovine, and 36 porcine isolates were screened to verify proper serovar typing belonging to *Salmonella* Typhimurium. Briefly, samples were streaked for isolation onto blood agar plates containing 5% sheep blood and incubated overnight at 37 °C. A single colony was first tested with the *Salmonella* O Antiserum group Poly A-I, & Vi, and then *Salmonella* O Antiserum Group B, factors 1, 4, 5, 12 (BD Diagnostic Systems, Fisher Scientific, Hampton, NH, USA). After antibody confirmation, the *Salmonella* Typhimurium isolates were grown in 1 mL of trypticase soy broth (TSB), and generated stocks were frozen at −80 °C in 10% sterile glycerol.

### 2.2. Integron and AMR Testing

A portion of each *Salmonella* Typhimurium stock isolate was scraped into a separate microcentrifuge tube, thawed, and centrifuged for 5 min at 5000× *g*. The supernatant was removed, and each pellet was resuspended in molecular grade water in a 1:3 ratio (10 µL cell pellet suspended in 30 µL of water). A total of 5 µL of each washed, resuspended isolate was used as a template and added to the following PCR mastermix for a 25-µL total reaction volume: 2.5 µL 1× Amplitaq Gold Buffer II and 1.5 mM MgCl_2_ (Applied Biosystems, Foster City, CA, USA), 0.8 mM dNTPs (0.2 mM each) (Roche Applied Sciences, Indianapolis, IN, USA), 0.4 µM of each primer (Int forward primer sequence: 5′-GGC ATC CAA GCA GCA AGC-3′; Int reverse primer sequence: 5′-AAG CAG ACT TGA CCT GAT-3′), 1.875 U Amplitaq Gold polymerase (Applied Biosystems, Foster City, CA, USA), and 2.5 µL 5× Q-Solution (Qiagen, Valencia, CA, USA).

The primers amplify the variable region between the 5′CS to 3′CS region of class 1 integrons [9]. Each reaction was overlaid with 30 µL of Chill Out wax (Bio-Rad, Hercules, CA, USA) to prevent evaporation and placed into an MJ Research 60 place thermal cycler (Bio-Rad). Thermal cycling conditions consisted of an initial incubation at 94 °C for 10 min to activate the polymerase and lyse cells, followed by 35 cycles of 94 °C for 30 s, 54 °C for 1 min, 72 °C for 1.5 min, and a final extension incubation at 72 °C for 10 min.

PCR products were analyzed by agarose gel electrophoresis using the FlashGel^®^ DNA System (Lonza Group, Ltd., Basel, Switzerland) and visualized by UV light transillumination. A 100 bp–4 kb molecular weight marker (Lonza Group, Ltd., Basel, Switzerland) was concordantly run on the gel as a ladder to aid in the calculation of the size of the amplified DNA fragments. A positive control sample generated from purified DNA from two isolates previously analyzed [9] for class 1 integrons and containing integron sizes of 1000, 1200, and 1600 was included (5 pg total) with each PCR and gel. Samples containing integron sizes of 1000, 1200, 1600, 1800, or both 1000 + 1200 bp were recorded and subsequently re-run on a 1% agarose gel containing a marker and a positive control for proper band size identification. Integron bands were excised from the gel and submitted for DNA purification using the QIAquick PCR Purification kit (Qiagen, Hilden, Germany).

All *Salmonella* Typhimurium isolates used in this study were tested for susceptibility to 16 antimicrobial agents by the disk diffusion assay according to CLSI standard procedures. The AMR testing panel consisted of the following sixteen antimicrobial drugs—amoxicillin–clavulanate (AMC-30), cephalothin (CF-30), chloramphenicol (C-30), ampicillin (AM-10), ceftiofur (CTO-30), enrofloxacin (ERF-5), streptomycin (S-10), triple sulfa (SSS-0.25), tetracycline (TE-30) sulfamethoxazole/trimethoprim (SXT 23.75–1.25), cefoxitin (FOX-30), ciprofloxacin (CIP-5), florfenicol (FFC-30), gentamicin (GM-10), kanamycin (K-30), and nalidixic acid (NA-30). *Escherichia coli* (*E. coli*) ATCC 25922 and *Staphylococcus aureus* ATCC 25923 were used as quality controls.

### 2.3. Isolate Growth and Extraction for Proteomic and Metabolomics Profiling

Nine *S.* Typhimurium isolates (three human, three porcine, and three bovine) were selected to undergo an antimicrobial drug growth challenge followed by a non-targeted metabolomics analysis. Criteria for selection were the presence of both 1000 and 1200 base pair integrons, and matching susceptibility/resistance profiles across the 16 drugs tested. Five drugs, ampicillin, chloramphenicol, streptomycin, sulfisoxazole, and tetracycline (ACSSuT panel; Sigma Aldrich, St. Louis, MO, USA), were selected for the *S.* Typhimurium antimicrobial drug challenge.

Ampicillin, chloramphenicol, tetracycline, and streptomycin were each dissolved in water to the desired stock concentration. Sulfisoxazole was added to 10% HCl and heated at 80 °C until dissolved. The sulfisoxazole–acid mix was added to TSB, the broth was neutralized to pH 7.0 using NaOH, and the other antibiotics were subsequently added. The final concentration of each antibiotic was based on the recommended minimum inhibitory concentration (MIC) recommended by the Clinical Laboratory and Standards Institute, as shown in Table 1.

Isolates were processed using standard laboratory procedures. They were thawed and streaked for isolation on sheep blood agar plates. One resulting colony from each selected isolate was suspended in 0.5 mL of TSB; 100 µL was inoculated into 20 mL of normal TSB (no drug = ND) and 100 µL was inoculated into 20 mL of ACSSuT TSB (Drug = D). The only difference between the ND and D was that the ND group of cultures were without antimicrobials. Cultures were then incubated with shaking at 37 °C for 24 h. After pelleting at 4300× *g* for 10 min at 4 °C and supernatant removal, the wet weight of each culture pellet was recorded and adjusted to 20 mg. Pellets were washed with phosphate-buffered saline (PBS) and centrifuged again as above; after discarding PBS supernatant, the pellets were frozen at −20 °C. Each sample pellet was thawed at 4 °C, suspended in methyl tert-butyl ether (MTBE), and sonicated for 30 s intervals for a total of 6 cycles, with a 30 s cooling on ice between cycles. The sonicated lysates were then centrifuged at 2500× *g* for 5 min at 4 °C, and 150 µL of LC-MS grade water and an additional 100 µL MTBE was added to the cleared supernatants. After sealing with Parafilm, sample tubes were vortexed at room temperature for 15 min, incubated at −80 °C for 15 min, and centrifuged at 15,890× *g* for 15 min at 4 °C. Samples were then divided by a non-polar supernatant, a polar supernatant, and protein lysates. Each layer was dried via nitrogen gas and stored at −80 °C for metabolomics analysis.

### 2.4. Metabolomic Profiling by UPLC- and GC–MS

An ultra-performance liquid chromatography–mass spectrometry (UPLC-MS) analysis was performed on a Waters Xevo G2-TOF MS coupled with a Waters Acquity UPLC [10]. Separation was performed on a UPLC T3 reverse phase column and data were collected in MSE mode (alternating low and high collision energy) [11]. For the gas chromatography–mass spectrometry (GC–MS) analysis, cell extracts were dried and derivatized using a standard protocol. Briefly, GC–MS data were acquired on a Thermo Scientific Trace-ISQ GC–MS system (Waltham, MA, USA) with separation using a 30 m TG-5MS column. Data from both UPLC-MS and GC–MS acquisitions were processed using XCMS (https://www.bioconductor.org/packages/release/bioc/html/xcms.html, accessed on 31 March 2022) for peak detection, retention time alignment, and normalization [12]. Metabolite annotation of GC–MS data was performed by grouping molecular features into peak groups using AMDIS software (http://www.amdis.net/, accessed on 31 March 2022) and screening spectra against the CSU in-house spectral library, NIST GC–MS spectral library, and the Golm Metabolite Database (http://gmd.mpimp-golm.mpg.de/, accessed on 31 March 2022). Annotations of UPLC-MS data were performed by an unbiased grouping of molecular features into spectra based on correlational clustering across the dataset [10] and screening spectra against the CSU in-house spectral library (consisting of approximately 1100 compounds), NIST LC-MS spectral library, and MassBank spectral library [12].

### 2.5. Statistical Analysis (MetaboAnalyst 4.0)

Data analysis of the biomarkers was completed using MetaboAnalyst 4.0 (MetaboAnalyst 4.0 is available at https://www.metaboanalyst.ca/ (accessed on 26 April 2021) and its R packages are available at https://github.com/xia-lab/MetaboAnalystR, (accessed on 26 April 2021)). The UPLC and GC–MS spectra were combined, normalized, and scaled. To determine the statistically significant (S.S.) metabolites, a pairwise analysis was conducted, including a non-parametric Wilcoxon rank-sum test and fold-change analysis. A two-way analysis of variance (ANOVA) followed by a principal component analysis (PCA) and heatmapping were used to determine and visualize the species and drug effects and interactions. A pathway analysis was then conducted to match S.S. metabolites to known metabolic pathways and determine the biological significance of those pathways.

Multiple features of this program were used, including “Two-factor”, “Statistical Analysis”, and “Pathway Analysis”, to conduct multiple statistical tests, including Wilcoxon rank-sum, fold-change, two-way ANOVA, PCA, and heatmapping. Conducting the “Pathway Analysis” in MetaboAnalyst required all metabolites to have an HMDB identifier. The Human Metabolome Database (HMDB) is a website that compiles detailed information about metabolites and their roles in human metabolic pathways and assigns HMDB identifiers or numbers.

## 3. Results

### 3.1. AMR Patterns and Integrons

The most common AMR pattern among all resistant samples (23/126 = 18.3%) was ampicillin, amoxicillin–clavulanate, streptomycin, sulfonamides, tetracycline, chloramphenicol, and florfenicol (coded as AMC-AM-S10-SSS-TE-C-FFC). All isolates with this AMR pattern carried both the 1000 and 1200 bp integrons.

### 3.2. Metabolite Expression by Drug Treatment and Host Species

**Figure 1:**
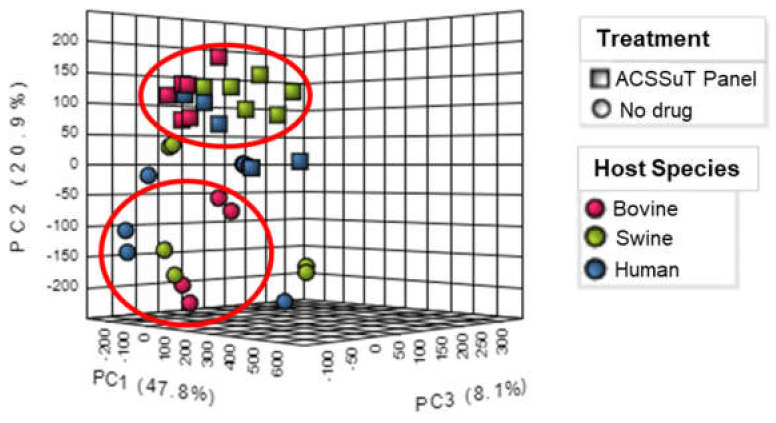
PCA chart derived from two–way ANOVA showing clustering of samples by drug treatment.

**Figure 2:**
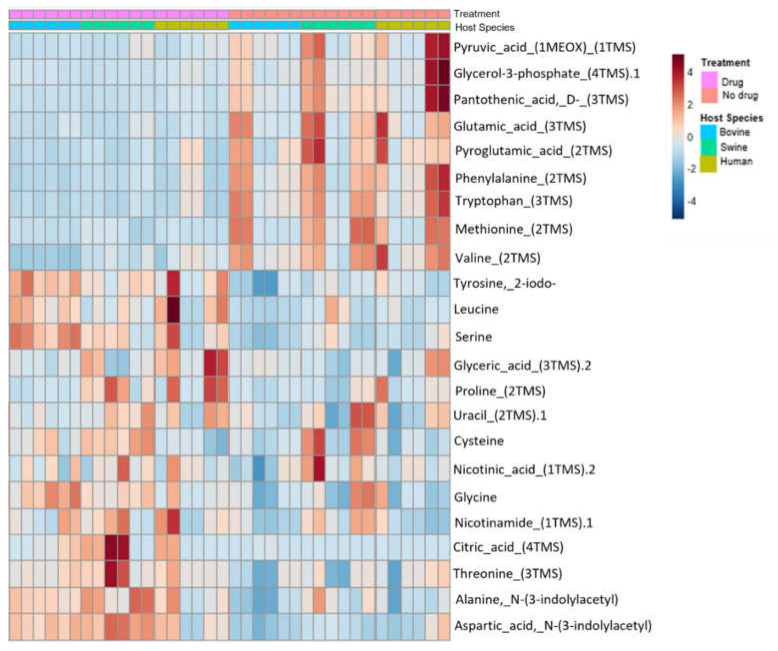
Heatmap derived from two–way ANOVA showing clustering of metabolite concentrations based on drug treatment.

Visualization by the principal component analysis (Figure 1) and the two-way ANOVA heatmap (Figure 2) showed that a greater effect on metabolite production was apparent when the samples were exposed to the full drug (ACSSuT panel) treatment, irrespective of species.

### 3.3. Metabolite Expression and Matched Metabolic Pathways

Wilcoxon rank-sum showed 653 metabolites that had an S.S. concentration difference (59.69% increased and 40.31% decreased expressions) when the sample was exposed to the ACSSuT antibiotic panel versus when it was not. Of those 653 metabolites, 23 unique metabolites were annotated by the PMF, identifiable by HMDB, and matched to one or multiple of the 9 statistically significant metabolic pathways in MetaboAnalyst. Of these, 60.87% of metabolites had an increased expression when exposed to antibiotics and 39.13% had a decreased expression (Figure 2).

Methionine, nicotinamide, nicotinate, pantothenate, phenylalanine, proline, pyroglutamic acid, pyruvate, serine, threonine, tryptophan, tyrosine, uracil, and valine significantly increased with full drug treatment. Conversely, alanine, aspartate, citrate, cysteine, glutamate, glycerate, glycerone phosphate, glycine, and leucine decreased with full drug treatment.

The metabolic pathways matched to the significantly different metabolites include glycine, serine, and threonine metabolism; alanine, aspartate, and glutamate metabolism; aminoacyl-tRNA biosynthesis; pantothenate and CoA biosynthesis; glutathione metabolism; valine, leucine, and isoleucine biosynthesis; nicotinate and nicotinamide metabolism; glyoxylate and dicarboxylate metabolism; and beta-Alanine metabolism, in order of descending pathway impact scores (Table 2).

### 3.4. Univariate Analysis

A between-subject, two-way ANOVA identified 297 metabolites (Table 3) that were statistically significant only for the treatment factor. No metabolites were found to be significant for the host species factor or the interaction between host species and treatment.

## 4. Discussion

In this study, we investigated metabolite expression patterns in AMR *Salmonella* Typhimurium isolated from human, bovine, and swine when exposed to antibiotics. We were able to demonstrate a greater difference in metabolite expression when the isolates were exposed to the full drug challenge compared to no drug exposure, irrespective of host species. The univariate analysis further confirmed that metabolite expression changes were significant only according to the treatment factor, not according to the host species or interaction of the host species and treatment. Metabolite expression being non-host specific suggests that AMR *Salmonella* Typhimurium drug targets are consistent across human, bovine, and swine hosts. This finding has great significance when considering that future drug testing on AMR *Salmonella* Typhimurium in swine and bovine could be translated to human treatments.

While the expression of 23 specific metabolites significantly changed when exposed to the full drug treatment and these upregulated metabolites each matched significant metabolic pathways, a specific resistance mechanism remains unclear. These isolates were exposed to multiple antimicrobial drugs and each drug has a different mechanism of action. Therefore, there are potentially many mechanisms of resistance that have developed in these isolates [4]. As per Hoerr et al. (2016), the metabolic profiles could be separated in a fingerprint, and based on the specific fingerprints obtained for different classes of antibiotics, the mode of action of several antibiotics could be predicted. The profiles could also be used as potential drug targets for pharmaceutical companies. Over the past few decades, there has been a decline in approvals of new antibiotic drugs in the market by the US Food and Drug Administration (FDA) [13,14]. The number of new antibiotics being developed every year decreases due to the challenges of effectively dispatching both antibiotic-resistant bacteria and novel infectious bacteria [15]. If we want to reverse these trends and facilitate new approaches to overcoming resistance, we must first understand the microbial forces responsible for developing resistance [16]. Metabolomics in particular offers a unique strategy to detect metabolic changes that occur in an organism in response to drugs and the outcomes of such studies can provide insights into their corresponding modes of action [17,18].

The significant changes observed in our study include increases in methionine, nicotinamide, nicotinate, pantothenate, phenylalanine, proline, pyroglutamic acid, pyruvate, serine, threonine, tryptophan, tyrosine, uracil, and valine, and decreases in alanine, aspartate, citrate, cysteine, glutamate, glycerate, glycerone phosphate, glycine, and leucine. These metabolites were matched to nine significant metabolic pathways, including glycine, serine, and threonine metabolism; alanine, aspartate, and glutamate metabolism; aminoacyl-tRNA biosynthesis; pantothenate and CoA biosynthesis; glutathione metabolism; valine, leucine, and isoleucine biosynthesis; nicotinate and nicotinamide metabolism; glyoxylate and dicarboxylate metabolism; and beta-Alanine metabolism.

Lin et al. (2019) [19] found biosynthesis of amino acids, biosynthesis of phenylpropanoids, and purine metabolism were commonly enriched in MDR strains of *E. coli*, and the results concurred that antibiotic resistance affects the metabolite profiles of MDR bacteria. Several related metabolites, such as glycerol, were increased in MDR strains, while citric acid and succinic acid were decreased in MDR strains [19].

An established resistance mechanism against β-lactams, such as ampicillin, includes the production of metallo-β-lactamases, which inactivate the drug through a cleavage process. The metallo-β-lactamases are especially threatening due to their ability to inactivate multiple β-lactams and their insensitivity to β-lactamase inhibitors that target the acyl serine transferases. This resistance mechanism has been identified in extended-spectrum β-lactamases where two amino acid substitutions are critical, a serine-for-arginine and a lysine-for-glutamate [20]. This substitution may explain the increased expression of serine and the decreased expression of glutamate observed when isolates are exposed to the ACSSuT drug panel in our study. Aspartate has also been identified as a critical component of the metallo-β-lactamases; thus, the increased expression of aspartate may support this mechanism [20].

Perhaps one of the largest resistance mechanisms is through decreasing TCA cycle flux. Previous studies have shown that exogenous alanine and/or glucose increase susceptibility to antibiotic treatment by increasing TCA flux and thereby increasing drug uptake by the cell [21]. Therefore, it is possible that decreased TCA flux could contribute to decreased drug susceptibility. Decreased concentrations of pyruvate and glutamate in our study support this conclusion, as pyruvate directly feeds the TCA cycle and glutamate is converted to pyruvate by α-ketoglutarate [22].

These data from our study suggest that another resistance mechanism utilized by these AMR isolates may be initiated from the aminoacyl-tRNA pathway. Aminoacyl-tRNA biosynthesis is responsible for changing cell membrane properties and increasing a pathogen’s resistance. It has previously been identified as an attractive drug target [22]. This pathway likely acts by decreasing cell permeability and, thus, inhibiting drug entrance into the cell. The aminoacyl-tRNA biosynthesis pathway in our study is significantly altered when isolates are exposed to the ACSSuT antibiotic panel.

Alanine is a required component of cell wall peptidoglycan and it has been demonstrated that inhibition of alanine transport results in increased susceptibility to drugs [23]. Increased concentrations of alanine may indicate that the cell wall has undergone peptidoglycan remodeling, resulting in decreased susceptibility.

In our study, citrate (citric acid) has the highest fold change of any of the metabolites matched to a significant pathway, but its possible role in antimicrobial resistance is less clear. Citrate has previously been described as having a role in the regulation of cell division and gene expression and is known to be a chelator, which may allow bacteria to manage intracellular concentrations of cations. Previous research has shown an increase in citrate concentrations when *Salmonella* aureus is exposed to cold temperatures, as well as upregulated cell division proteins [24]. Therefore, increased citrate concentrations may suggest that *S.* Typhimurium depends on this metabolite to maintain intracellular Ca++ concentrations and increases the rate of cell division. An increased rate of cell division would also increase the chances of DNA mutation occurring and antibiotic resistance developing. Further examining the role of citrate in bacterial survival and AMR is warranted.

In future research, exposing isolates to only one antibiotic or one class of antibiotics would allow for a more specific interpretation of the expressed metabolites and potentially provide more robust evidence on resistance mechanisms. Interpretation of these data is limited due to the multiple mechanisms by which the ACSSuT panel targets bacteria. Resistance mechanisms against one class of antibiotics differ from those against another class, hence why bacteria resistant to one class may be susceptible to a different one [25]. This explains why a distinct resistance mechanism was not identifiable in this project. Exposure to a single antibiotic class may create a more easily identifiable profile of metabolites attributable to a specific resistance mechanism.

## 5. Conclusions

The findings of this study suggest that exposing AMR *Salmonella* Typhimurium to an ACSSuT panel significantly alters metabolic pathways and, thus, metabolite expression by the bacteria. This research supports the continuation of using metabolomics to study AMR and identify resistance mechanisms, which could become future drug or testing targets. However, further studies are necessary to identify specific resistance mechanisms for different classes of antibiotics.

## Figures and Tables

**Table 1:** Recommended MIC values for *Salmonella enterica* serotype Typhimurium for the ACSSuT pattern according to 2014 CLSI standards.

Drug Panel	MIC
Ampicillin	32 µg/mL
Chloramphenicol	32 µg/mL
Streptomycin	64 µg/mL
Sulfisoxazole	512 µg/mL
Tetracycline	16 µg/mL

**Table 2:** Significant metabolites from the Wilcoxon rank-sum test with the metabolic pathway and the associated fold changes observed across host species.

					All Hosts			Bovine			Swine			Human		
Metabolic Pathway	Impact	*p*-Value	FDR	Metabolite	Fold Change (FD/ND)	*p*-Value	FDR	Fold Change (FD/ND)	*p*-Value	FDR	Fold Change (FD/ND)	*p*-Value	FDR	Fold Change (FD/ND)	*p*-Value	FDR
Aminoacyl-tRNA biosynthesis	0.2	1.93 × 10^−7^	1.66 × 10^−5^	Phenylalanine	0.565	0.0000581	0.00043499	0.433	0.0021645	0.0096991	0.737			0.535		
				Cysteine	1.515	0.0096309	0.023444	0.118	0.0021645	0.0096991	0.228	0.0021645	0.022605	1.400		
				Glycine	1.858	0.00000394	0.0000655	2.003	0.0021645	0.0096991	1.812			1.723		
				Aspartate	3.077	3.06 × 10^−8^	0.00000283	3.174	0.0021645	0.0096991	3.883	0.0021645	0.022605	2.178		
				Serine	2.837	8.22 × 10^−8^	0.00000426	4.469	0.0021645	0.0096991	1.755	0.0021645	0.022605	2.240		
				Methionine	0.594	0.020464	0.042605	0.498	0.0021645	0.0096991	0.588			0.708		
				Valine	0.675	0.0064022	0.017165	0.391	0.0021645	0.0096991	0.925			0.782		
				Alanine	2.065	3.06 × 10^−8^	0.00000283	2.149	0.0021645	0.0096991	2.240	0.0021645	0.022605	1.910		
				Leucine	0.087	0.0023492	0.0080271	0.106	0.015152	0.041482	0.063	0.0021645	0.022605	0.116		
				Threonine	2.233	2.2 × 10^−10^	0.000000285	2.065	0.0021645	0.0096991	3.156	0.0021645	0.022605	1.834		
				Tryptophan	2.143	0.0000164	0.00017127	1.789	0.0021645	0.0096991	2.671			1.647		
				Tyrosine	2.183	1.48 × 10^−8^	0.00000212	2.932	0.0021645	0.0096991	2.054	0.0021645	0.022605	1.959		
				Proline	2.150	0.00075777	0.0033152	1.153			3.432			2.041		
				Glutamate	0.321	0.0000239	0.00021944	0.353			0.497			0.243		
Pantothenate and CoA biosynthesis	0.144	0.00304	0.10686	Pantothenate	0.414	0.000000	0.000005	0.440	0.0021645	0.0096991	0.389	0.0021645	0.022605	0.429		
				Valine	0.675	0.006402	0.017165	0.391	0.0021645	0.0096991	0.925			0.782		
				Aspartate	3.077	0.000000	0.000003	3.174	0.0021645	0.0096991	3.883	0.0021645	0.022605	1.300		
				Cysteine	1.515	0.009631	0.023444	0.118	0.0021645	0.0096991	0.228	0.0021645	0.022605	1.400		
				Pyruvate	0.458	0.002642	0.008841	0.382	0.008658	0.026381	1.570	0.0021645	0.022605	0.394		
				Uracil	1.477	0.020464	0.042605	0.977			1.848			1.474		
Glycine, serine, and threonine metabolism	0.456	0.00373	0.10686	Serine	2.837	0.000000	0.000004	4.469	0.0021645	0.0096991	1.755	0.0021645	0.022605	2.240		
				Glycine	1.858	0.000004	0.000066	2.003	0.0021645	0.0096991	1.812			1.723		
				Aspartate	3.077	0.000000	0.000003	3.174	0.0021645	0.0096991	3.883	0.0021645	0.022605	1.300		
				Glycerate	1.691	0.000503	0.002423	0.631	0.008658	0.026381	2.225			2.473		
				Threonine	2.233	0.000000	0.000000	2.065	0.0021645	0.0096991	3.156	0.0021645	0.022605	1.834		
				Pyruvate	0.458	0.002642	0.008841	0.382	0.008658	0.026381	1.570	0.0021645	0.022605	0.394		
				Tryptophan	2.143	0.000016	0.000171	1.789	0.0021645	0.0096991	2.671			1.647		
Glutathione metabolism	0.118	0.01061	0.2144	Glycine	1.858	0.000004	0.000066	2.003	0.0021645	0.0096991	1.812			1.723		
				Cysteine	1.515	0.009631	0.023444	0.118	0.0021645	0.0096991	0.228	0.0021645	0.022605	1.400		
				Pyroglutamic Acid	0.679	0.005177	0.014605	0.613			0.748			0.693		
				Glutamate	0.321	0.000024	0.000219	0.353			0.497			0.243		
Nicotinate and Nicotinamide metabolism	0.066	0.0125	0.2144	Aspartate	3.077	0.000000	0.000003	3.174	0.0021645	0.0096991	3.883	0.0021645	0.022605	1.300		
				Glycerone phosphate	0.582	0.000001	0.000027	0.644	0.004329	0.016886	0.579	0.0021645	0.022605	0.536		
				Nicotinamide	1.858	0.000245	0.001366	1.505			1.666			2.495		
				Nicotinate	1.435	0.000007	0.000098	1.587	0.008658	0.026381	1.455			1.832		
Glyoxylate and dicarboxylate metabolism	0.055	0.0269	0.38631	Citrate	7.934	0.000024	0.000219	2.799			20.017			6.503		
				Glycerate	1.691	0.000503	0.002423	0.631	0.008658	0.026381	2.225			2.473		
				Glycine	1.858	0.000004	0.000066	2.003	0.0021645	0.0096991	1.812			1.723		
				Glutamate	0.321	0.000024	0.000219	0.353			0.497			0.243		
				Serine	2.837	0.000000	0.000004	4.469	0.0021645	0.0096991	1.755	0.0021645	0.022605	2.240		
				Pyruvate	0.458	0.002642	0.008841	0.382	0.008658	0.026381	1.570	0.0021645	0.022605	0.394		
beta-Alanine Metabolism	0	0.0458	0.43434	Aspartate	3.077	0.000000	0.000003	3.174	0.0021645	0.0096991	3.883	0.0021645	0.022605	1.300		
				Pantothenate	0.414	0.000000	0.000005	0.440	0.0021645	0.0096991	0.389	0.0021645	0.022605	0.429		
				Uracil	1.477	0.020464	0.042605	0.977			1.848			1.474		
Valine, leucine, and isoleucine biosynthesis	0.107	0.0475	0.43434	Threonine	2.233	0.000000	0.000000	2.065	0.0021645	0.0096991	3.156	0.0021645	0.022605	1.834		
				Leucine	0.087	0.002349	0.008027	0.106	0.015152	0.041482	0.063	0.0021645	0.022605	0.116		
				Pyruvate	0.458	0.002642	0.008841	0.382	0.008658	0.026381	1.570	0.0021645	0.022605	0.394		
				Valine	0.675	0.006402	0.017165	0.391	0.0021645	0.0096991	0.925			0.782		
Alanine, aspartate and glutamate metabolism	0.45	0.0475	0.43434	Aspartate	3.077	0.000000	0.000003	3.174	0.0021645	0.0096991	3.883	0.0021645	0.022605	1.300		
				Alanine	2.065	0.000000	0.000003	2.149	0.0021645	0.0096991	2.240	0.0021645	0.022605	1.910		
				Glutamate	0.321	0.000024	0.000219	0.353			0.497			0.243		
				Pyruvate	0.458	0.002642	0.008841	0.382	0.008658	0.026381	1.570	0.0021645	0.022605	0.394		

FDR = false discovery rate; FDR helps control for falsely positive significant features; FDR < 0.05 has less than a 5% probability of being a falsely significant feature. Fold change of > 1 indicates an increase in metabolite expression when exposed to full drug treatment and a fold change of <1 indicates a decrease in metabolite expression when exposed to the full drug treatment. 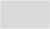 Non-significant metabolites.

**Table 3:** Significant identifiable metabolites found via univariate, between-subject, two-way ANOVA.

Metabolite	*p*-Value	FDR
2-Piperidinecarboxylic_acid_1MEOX_2TMS	0.00958	0.04464
Adenine_1TMS	0.00275	0.02104
Alanine, *N*-3-indolylacetyl	0.00002	0.00135
Aspartic acid, *N*-3-indolylacetyl	0.00000	0.00032
Butanoic acid,_3-hydroxy-0.2	0.00064	0.00847
Butanoic_acid, 4-hydroxy-_2TMS	0.00035	0.00576
Cinnamic_acid, 2-hydroxy-, trans-	0.000003	0.00040
Cohibin_A.1	0.00374	0.02508
Coixenolide_2	0.00869	0.04154
Coixenolide_4	0.00561	0.03144
Cysteamine_3TMS	0.00531	0.03071
Cysteine_3TMS	0.01144	0.04989
Glycerol-3-phosphate_4TMS.2	0.00118	0.01276
Glycine	0.00004	0.00173
Guanosine	0.00001	0.00088
Guanosine,_2′-deoxy-_4TMS.1	0.00027	0.00504
Guanosine_4TMS coeluting_with_Guanosine_5TMS	0.00261	0.02063
Iminodiacetic_acid_3TMS	0.00185	0.01715
Isoleucine_2TMS	0.00071	0.00914
Lactose	0.00001	0.00065
Leucine	0.00032	0.00541
Leucine,_cyclo-	0.00352	0.02428
Leucine_2TMS	0.00051	0.00704
Levulinic_acid	0.00057	0.00760
Luteolin	0.00474	0.02871
Naringenin	0.00066	0.00867
Oxamide_3TMS	0.00017	0.00383
Pantothenic_acid,_D-_3TMS	0.00799	0.04009
Phenylalanine_2TMS	0.00003	0.00147
Phosphomycin	0.000000008	0.00001
Pinitol,_D-_5TMS	0.00363	0.02488
Putrescine_4TMS	0.00224	0.01912
Pyridine	0.00443	0.02729
Pyridoxamine	0.00859	0.04136
Pyroglutamic_acid_2TMS	0.00976	0.04528
Quercetin	0.00003	0.00147
Serine	0.00000003	0.00001
Thiamine	0.00004	0.00173
Threitol,_dithio-	0.00166	0.01589
Thymidine-5′-monophosphoric-acid-3TMS	0.00020	0.00426
Tryptophan_3TMS	0.00007	0.00249
Tyrosine,_2-iodo-	0.000000017	0.00001
Tyrosine_3TMS	0.00001	0.00094
Uric_acid	0.00017	0.00383
Valine_2TMS	0.00008	0.00280
Xanthine_3TMS	0.00772	0.03903

FDR = false discovery rate; FDR helps control for falsely positive significant features; FDR < 0.05 has less than a 5% probability of being a falsely significant feature.

## Data Availability

All data generated or analyzed during this study are included in this published article.
